# A Novel Index Based on Binary Entropy to Confirm the Spatial Expansion Degree of Urban Sprawl

**DOI:** 10.3390/e20080559

**Published:** 2018-07-27

**Authors:** Zhen Chen, Yinkang Zhou, Xiaobin Jin

**Affiliations:** School of Geographic and Oceanographic Sciences, Nanjing University, Nanjing 210046, China

**Keywords:** urban sprawl, point pattern analysis, binary entropy, index

## Abstract

The phenomenon of urban sprawl has received much attention. Accurately confirming the spatial expansion degree of urban sprawl (SEDUS) is a prerequisite to controlling urban sprawl. However, there is no reliable metric to accurately measure SEDUS. In this paper, based on binary entropy, we propose a new index named the spatial expansion degree index (SEDI), to overcome this difficulty. The study shows that the new index can accurately determine SEDUS and, compared with other commonly used measures, the new index has an obvious advantage in measuring SEDUS. The new index belongs to the second-order metrics of point pattern analysis, and greatly extends the concept of entropy. The new index can also be applied to other spatial differentiation research from a broader perspective. Although the new index is influenced by the scaling problem, because of small differences between different scales, given that the partition scheme in the research process is the same, the new index is a quite robust method for measuring SEDUS.

## 1. Introduction

Urban sprawl is considered a threat to sustainable economic and social development in the world today [1]. It is reported that worldwide the built-up land area increased 58,000 km^2^ from 1970 to 2000 and, at this rate, by 2030, 1,527,000 km^2^ of built-up land will be added globally [2]. Meanwhile, the built-up land expansion often encroaches on suburban peripheries and results in cropland fragmentation [3,4], so its spatial form mostly appears ‘‘dispersed’’ [5]. The phenomenon of the high-speed and dispersed expansion of built-up land has become known as urban sprawl [5,6,7,8]. 

There are two different viewpoints about the impact of urban sprawl. One idea is that urban sprawl can relieve traffic congestion in urban central area, relax residential pressure, increase employment, and promote social fairness [5,9]. The opposite view is that development associated with urban sprawl not only decreases the amount of cultivated land and forest resources, endangers food and energy security, destroys the environment, and also affects biological diversity [1,10,11,12,13,14,15]. As time has passed, the phenomenon of urban sprawl has received much attention, and most scholars share the view that the negative aspects of the impact outweigh the positive ones [8].

Urban sprawl is considered a multidimensional phenomenon [5,8,16,17,18,19]. As the subject became popularized, some of the researchers began conceptualizing multifaceted analytical frameworks and establishing multiple indices by GIS analysis or descriptive statistical analysis to measure the sprawl [6,18,19,20]. Galster et al. conducted a pioneering study, which proposed eight dimensions—density, continuity, concentration, clustering, centrality, nuclearity, mixed uses, and proximity—to measure housing sprawl in 13 urbanized areas [18]. Tsai employed a set of four dimensions—size, density, degree of equal distribution and degree of clustering—to systematically identify a metropolitan form [20]; Hamidi and Ewing operationalized four dimensions—development density, land use mix, activity centering and street accessibility—to study the changes of urban sprawl between 2000 and 2010 in the United States [19]. However, if only considering urban sprawl as a typical geographical phenomenon and focusing solely on the external spatial form, two indispensable dimensions increasing amount of built-up land area and spatial expansion degree should be confirmed [7,21]. Measurement of increasing amount of built-up land area can be easily obtained by remote sensing imageries and techniques [8,13,22,23,24,25,26,27,28,29,30], however, there is no reliable metrics to measure the spatial expansion degree.

Accurately confirming the spatial expansion degree of urban sprawl (SEDUS) will not only provide monitoring information of land use/cover change and urban planning decision-making bases, but also make contributions to controlling urban sprawl [11,29,31,32] and the protection of the environment and ecosystems [7,29,30,31,32,33]. There are about 50 metrics that characterize the spatial degree of expansion of urban sprawl, and many of them have been implemented in the public domain statistical package FRAGSTATS3, which are generally known as spatial metrics or landscape metrics [6,20,34]. Spatial metrics have found important applications in quantifying SEDUS [6,16,20,23,31], however, many of them are correlated [6,34]. Often, different metrics employed may also result in contradictory conclusions [35]. 

Most of the spatial metrics used in the research of spatial heterogeneity require partitioning the study area, so partitioning methods can affect the values of the spatial metrics employed [14,29,34,36,37], which is the so-called modifiable area unit problem [29,36,37]. The problem includes two aspects: scale effect and zoning effect, respectively. The former refers to the phenomenon that the analytical results change with the aggregation of spatial data through different basic area units; the latter refers to the change caused by the effect of different aggregation methods on the same basic unit. Since scale effect to the spatial metrics varies from different metrics while the zoning effect has the commonness [36,37], the research in the influence of partition method on a new metric is often just focused on the scale effect [7,21,29,37].

**Figure 1:**
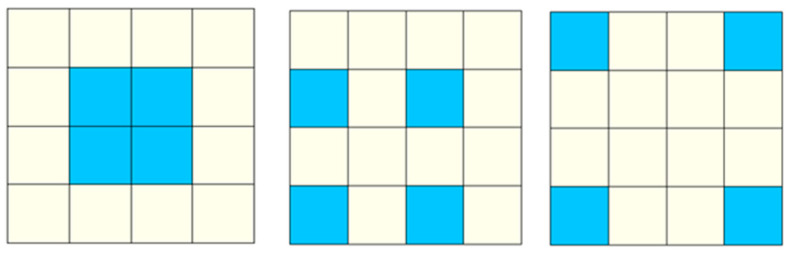
The limitations of Relative Shannon Entropy (RS) in describing the spatial expanding degree of urban sprawl (SEDUS). Note: This graph contains three scenarios, and each scenario contains 16 grid subzones or cells, where the blue grid cell area (1 × 1) indicates that the built-up land area is equal to the total area of the reporting subzone (the size of a cell is set to 1); every yellow subzone indicates the land without buildings in it. Obviously, the SEDUS of the three subplots from **left** to **right** is gradually deepening. As for RS, its value ranges from 0 to 1, and the greater the value is, the greater the SEDUS [6,29]. From the resulting values (the values are 0.5000), each zone has the same expansion degree.

Entropy methods, such as Shannon’s entropy and Renyi’s entropy [38], are perhaps the most widely used spatial metrics for measuring SEDUS with the integration of remote sensing and GIS [20,29,39,40,41,42,43]. In fact, the term of entropy is originally a thermodynamic item [44,45,46]. Claude Shannon introduced the concept of entropy into information theory and physics and provided a new scientific field [47]. Jaynes proposed the principle of maximum entropy on Shannon’s work to apply to subjective statistic inference; since then, entropy has occurred as a statistical inference method for supporting spatial locations [48,49]. Theil obtained a new understanding of Shannon’s entropy in the form of its relative value (i.e., relative entropy) and regarded it as a measure of a discrete probability distribution [50]. Yeh and Li used relative Shannon entropy (RS in the paper) to successfully measure the extent of urban sprawl. Henceforth, RS has become a main tool in measuring SEDUS [6,29,44]. However, for RS, there is still a considerable deficiency: built-up land with different configurations may have the same entropy value (Figure 1). That is, although RS is more robust than the other measures, developing new tools and metrics are highly demanded. Like many other spatial metrics, the modifiable area unit problem also exists in entropy methods [29].

In this paper, a new index named spatial expansion degree index (SEDI) is proposed to confirm the spatial expansion degree of urban sprawl. The structure of this paper is as follows: first, based on choosing the spatial analysis method and determining the dimensions of SEDUS, we elaborate on the building-up process of the new index and introduce the study area. Second, based on the case result, we discuss the obvious advantage of the new index compared with other commonly used measures, outline the characteristics of the new index, investigate the influence of partition method on the index, and propose its relativity in measuring SEDUS and future research directions. Finally, we provide our conclusions. 

## 2. Materials and Methods 

### 2.1. Building Spatial Expansion Degree Index (SEDI)

#### 2.1.1. Choosing the Spatial Analysis Method and Determining Dimensions of SEDUS 

In the building-up process of the new index, we adopted the second-order metrics of point pattern analysis as our spatial analysis method [51]. The so-called second-order metrics are also known as distance-based point pattern measures, which take an object of interest at a particular location in the study region as a point pattern and use the distances between points to characterize the geospatial object of interest [51]. That is, the spatial analysis method is on the basis of point-pair distance [51,52,53]. 

In this paper, by reference to partition methodology of the Geographical Analysis Machine (GAM) [52], we laid out a two-dimensional grid over the study region as a partitioning method, treated each grid centroid as the center of the subzone, and attached the message of the built-up land area in the corresponding subzone to each point (grid centroid). In this way, we set up a one-to-one correspondence relationship between the built-up land in the subzone and each grid centroid in the point pattern, that is, the term “two linked subzones” is equivalent to a “point-pair” in meaning. In addition, for the sake of convenient research, the built-up process of the new index goes from one point-pair to multiple point-pairs step-by-step.

**Figure 2:**
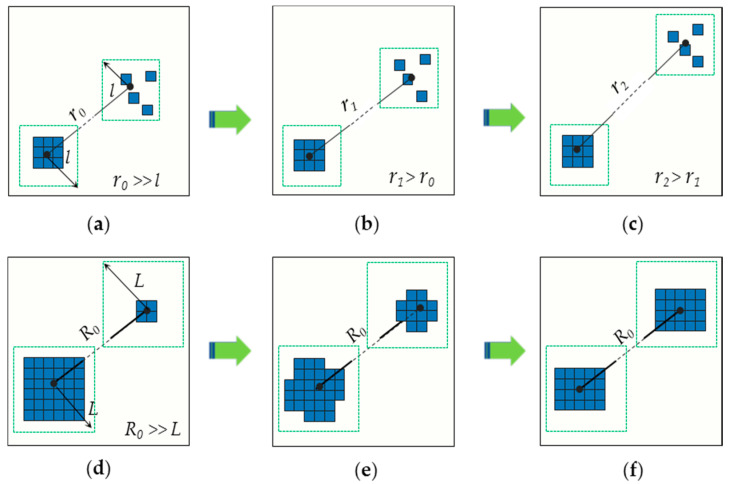
Spatial expanding degree of urban sprawl (SEDUS) is a function of the distances between point-pairs and built-up land area configurations in subzones. Note: Each blue grid cell can be understood as the basic unit of built-up land area, and each dotted green square box denotes the basic partition (i.e., subzone). (**a**–**c**) denote two linked subzones with constant built-up land area configurations and different distances, which characterizes the process that the SEDUS is gradually deepening with increasing distances. (**d**–**f**) represent two linked subzones with different built-up land area configurations and the same distance, which reveals that the SEDUS grows greater in more balanced area configurations (the process could be viewed as a course that the area unit of built-up land was moved from one subzone to another).

To confirm the spatial expanding degree of urban sprawl (SEDUS), it is necessary to determine the dimensions of SEDUS. This paper is influenced by the ideal of Jaeger et al. work which focused only on the external spatial form of urban sprawl, regardless of the driving forces of urban sprawl [7,21]. As seen in Figure 2, the SEDUS changes with two dimensions: the distances between point-pairs and built-up land area configurations in subzones; intuitively, the greater the distances and the more balanced area configurations, the greater the SEDUS. That is, SEDUS is a function of the two dimensions.

#### 2.1.2. Defining the Point-Pair Entropy Distance

**Figure 3:**
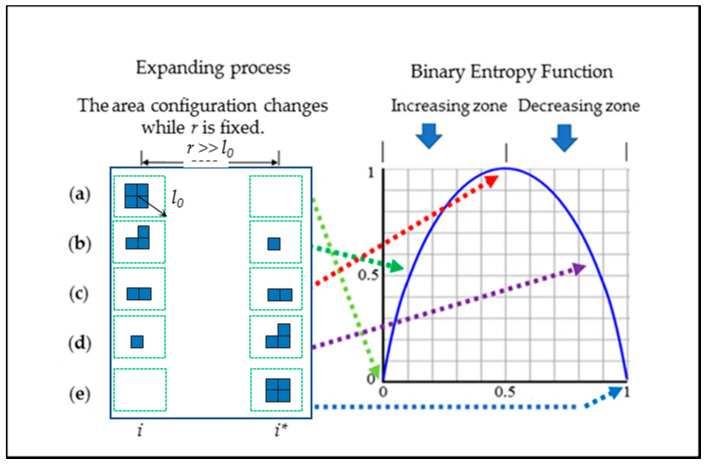
The diagram in the description of the correspondences between the binary entropy function and SEDUS in one point-pair configuration. Note: The legends in the figure are same as in Figure 2. Scenarios (**a**), (**b**), (**c**), (**d**), and (**e**), respectively, represent different SEDUS. Scenarios (**a**) and (**e**) display the lowest SEDUS, while Scenarios (**c**) displays the highest. Scenarios (**a**), (**c**) and (**e**) respectively correspond to the three special points of binary entropy function: the starting point, the midpoint, and the terminal point, respectively. Scenario (**b**) represents a random point in the changing process of the expansion from the lowest degree to the highest, and the process just corresponds to the increasing zone of the binary entropy function, while the scenario (**d**) displays just the opposite.

The task consists of two sides: how to measure the two dimensions of SEDUS and how to integrate the two dimensions in only one point-pair configuration. First, we utilized Pythagoras’ theorem (the straight-line distance between two points) [51] to measure the magnitude of the distance. Second, we employed binary entropy function (recorded as $B_{\left(i,i^{*}\right)}$) to measure the area configuration. $B_{\left(i,i^{*}\right)}$ is the shorthand form of binary entropy function, when $0\log_{2}0$ is taken, the value of $B_{\left(i,i^{*}\right)}$ is equal to *0*; when an even area configuration occurs, $B_{\left(i,i^{*}\right)}$ attains its maximum value [54]. These specific properties of $B_{\left(i,i^{*}\right)}$ match the process of the SEDUS well (Figure 3). Thirdly, we used the product (recorded as $ED_{\left(i,i^{*}\right)}$) of $B_{\left(i,i^{*}\right)}$ and the distances to characterize the comprehensive effect of SEDUS. The related mathematical expressions are written as:

(1) $$ED_{\left(i,i^{*}\right)}=kB_{\left(i,i^{*}\right)}r_{\left(i,i^{*}\right)}.$$

where:

(2) $$\;B_{\left(i,i^{*}\right)}=-P_{i}\log_{2}P_{i}-P_{i^{*}}\log_{2}P_{i^{*}}.$$

where:

(3) $$\begin{array}{c} Pi=p_{i}/\left(p_{i}+p_{i^{*}}\right);\\ P_{i^{*}}=1-Pi=p_{i^{*}}/\left(p_{i}+p_{i^{*}}\right). \end{array}$$

$ED_{\left(i,i^{*}\right)}$ is called point-pair entropy distance, which represents the SEDUS in only one point-pair configuration; *k* is a constant and is usually set to 1; $r_{\left(i,i^{*}\right)}$ represents the straight-line distance between two points; $p_{i}$ is the proportion of built-up land area in the *i*th subzone over the built-up land area in whole area ($p_{i}=x{}_{i}/\sum_{1}^{n}x{}_{i}$) [6,14,29]. From Equation (1), if the value of $r_{\left(i,i^{*}\right)}$ is fixed, the magnitude of $ED_{\left(i,i^{*}\right)}$ varies with the binary entropy function; when the expansion with a fully-even area configuration ($P_{i}=P_{i^{*}}$) occurs, the SEDUS reaches the highest value; given that the area configuration is fixed ($r_{\left(i,i^{*}\right)}$ is variable), the greater the value of $r_{\left(i,i^{*}\right)}$, the greater the magnitude of $ED_{\left(i,i^{*}\right)}$. Therefore, $ED_{\left(i,i^{*}\right)}$ well reflects the law that the SEDUS changes with its dimensions.

#### 2.1.3. Giving Weights to Point-Pair Entropy Distance and Optimizing $r_{\left(i,i^{*}\right)}$

**Figure 4:**
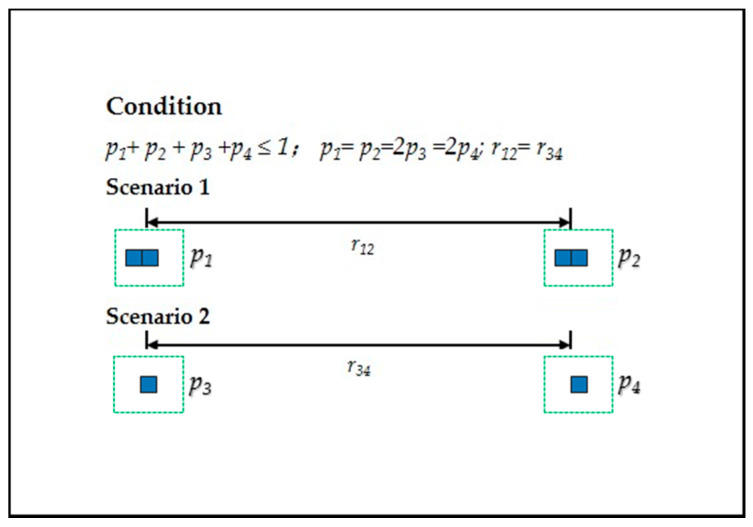
An example for illustrating the necessity of giving weights to point-pair entropy distances. Note: The legends in the figure are same as in Figure 2. Due to two scenarios with different built-up land area configurations ($p_{1}=p_{2}=2p_{3}=2p_{4}$) and the same distances ($r_{12}=r_{34}$), SEDUS in Scenario 1 is twice of that in Scenario 2. That is, giving no weight to point-pair entropy distance (other things being equal), when applying Equation (1) to the two scenarios, the resulting values of $ED_{\left(i,i^{*}\right)}$ are the same because $P_{i}=P_{i^{*}}$ (see Equation (3)). However, when we operated the adjusted $ED_{\left(i,i^{*}\right)}$ (Equation (4)), we found that the resulting values aligned with the SEDUS in the two scenarios.

Based on the point-pair entropy distance, we discuss the SEDUS with its dimensions in more than two point-pair configurations. First, as can be seen from Figure 4, if two point-pairs in a configuration occur, binary entropy function is incapable of describing this change. We also found in Figure 4 that, other things being equal, the greater the value of the sum of the proportion of the built-up land area, the higher the spatial expansion degree. To express this effect, we took $Y_{(}{}_{i,i^{*})}$ (recorded as $Y_{(}{}_{i,i^{*})}=p_{i}+p_{i^{*}}$) as the weight of $B_{\left(i,i^{*}\right)}$, and named the adjusted $ED_{\left(i,i^{*}\right)}$ as the weighted point-pair entropy distance. In fact, $Y_{(}{}_{i,i^{*})}$ is always equal to constant *1* in the only one point-pair configuration, thus, $ED_{\left(i,i^{*}\right)}$ can be regarded as a special case. The mathematical expression of the weighted point-pair entropy distance is written as:

(4) $$WED_{\left(i,i^{*}\right)}=kB_{\left(i,i^{*}\right)}Y_{\left(i,i^{*}\right)}r_{\left(i,i^{*}\right)}.$$

Second, let us investigate SEDUS with more than two points distances. In the case, the so-called “problem of monotonous reaction to increased spreading of three urban patches” will emerge [21]. This means that, when an urban area is broken up into more parts and the distribution of these parts becomes more dispersed, the SEDUS will increase monotonously as Figure 1 demonstrates.

**Figure 5:**
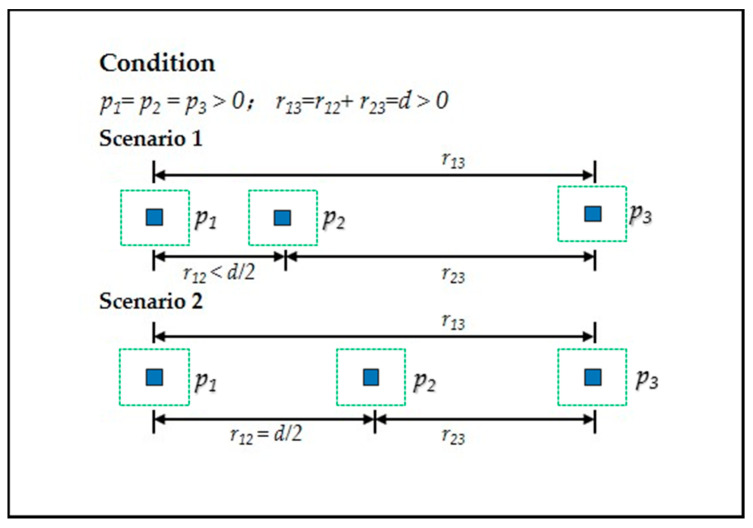
An example for illustrating the necessity of optimizing $r_{\left(i,i^{*}\right)}$ in configurations with three collinear subzones. The legends in the figure are same as in Figure 2. As the figure shows, three subzones with equal built-up land area line up, where the two outer subzones are fixed and the third one can freely shift along the line (Scenario 1); when the third subzone is right in the middle (Scenario 2), the SEDUS is the highest; in other words, the closer the third subzone shifts to one of the two outer subzones, the lower the SEDUS will be. However, when applying Equation (4) to the two scenarios, due to $p_{1}=p_{2}=p_{3}$ ($p_{i}$ is the proportion of built-up land area and does not refer to the patch’s code in the subzone) and $r_{13}+r_{12}+r_{23}=2d$, their resulting values are the same. Therefore, it is necessary to optimize $r_{\left(i,i^{*}\right)}$.

If computing SEDUS in more than a two-point configuration with no three being collinear, we can accomplish the task well by using the method of summing the resulting value of $WED_{\left(i,i^{*}\right)}$. However, when configurations with three points in a line occur, the method is powerless, as Figure 5 shows.

Jaeger et al. proposed a power function $f(x)=cx^{\gamma }$ (0<γ<1; *c* is a constant) to solve the problem (see [7]). We select the simplest square root function $f(x)=\sqrt{2x}$ to optimize $r_{\left(i,i^{*}\right)}$. Thus, Equation (4) is adjusted as:

(5) $$WED^{*}{}_{\left(i,i^{*}\right)}=kB_{\left(i,i^{*}\right)}Y_{\left(i,i^{*}\right)}\sqrt{2r_{\left(i,i^{*}\right)}}.$$

where $WED^{*}{}_{\left(i,i^{*}\right)}$ denotes the optimized weighted point-pair entropy distance; and $\sqrt{2r_{\left(i,i^{*}\right)}}$ denotes the optimized $r_{\left(i,i^{*}\right)}$. Simple verification shows that Equation (5) is effective. 

#### 2.1.4. Constructing Spatial Expansion Degree Index (SEDI)

Suppose a zone (the whole study area) with *n* subzones, if two subzones $\left(i,i^{*}\right)$ are randomly selected, n(n−1)/2 point-pairs in total can be obtained. We apply Equation (5) to all point-pairs and average all resulting values, the global average optimized weighted point-pair entropy distance can be developed as:

(6) $$D_{n}=2k\sum (B_{\left(i,i^{*}\right)}Y_{\left(i,i^{*}\right)}\sqrt{2r_{\left(i,i^{*}\right)}})/[n(n-1)].$$

Since Equation (6) contains an absolute amount, $\sqrt{2r_{\left(i,i^{*}\right)}}$, it is difficult to compare SEDUS in different regions. To solve the problem, by introducing the relative value, we further adjust Equation (6). The first work is to calculate the maximum of $D_{n}$. Since the partition scheme in the process of research remains the same, the point-to-point distances must be fixed. Meanwhile, when equal probability occurs within the zone (if a fully-even area configuration occurs, then $p_{i}=1/n$) (see [44]), $B_{\left(i,i^{*}\right)}$ reaches the maximum 1 (See Equations (2) and (3)), at the same time, $Y_{\left(i,i^{*}\right)}$ obtains the maximum 2/n, and $D_{n}$ obtains the maximum. The resulting maximum is written as:

(7) $$MAX(D_{n})=k(2/n)D_{n}=4k\sum \sqrt{2r_{\left(i,i^{*}\right)}}/[n^{2}(n-1)].$$

Then, SEDI is defined as the form of the relative value:

(8) $$SEDI=D_{n}/MAX(D_{n})=n\sum W_{\left(i,i^{*}\right)}\sqrt{2r_{\left(i,i^{*}\right)}}/[2\sum \sqrt{2r_{\left(i,i^{*}\right)}}].$$

where:

(9) $$W_{\left(i,i^{*}\right)}=B_{\left(i,i^{*}\right)}Y_{\left(i,i^{*}\right)}=B_{\left(i,i^{*}\right)}(p_{i}+p_{i^{*}}).$$

$W_{\left(i,i^{*}\right)}$ is a weighted version of $B_{\left(i,i^{*}\right)}$ (see Equations (2) and (3)), which is only associated with $p_{i}$. The values of the index range from 0 to 1, and the greater the value is, the greater the SEDUS.

### 2.2. Study Case

**Figure 6:**
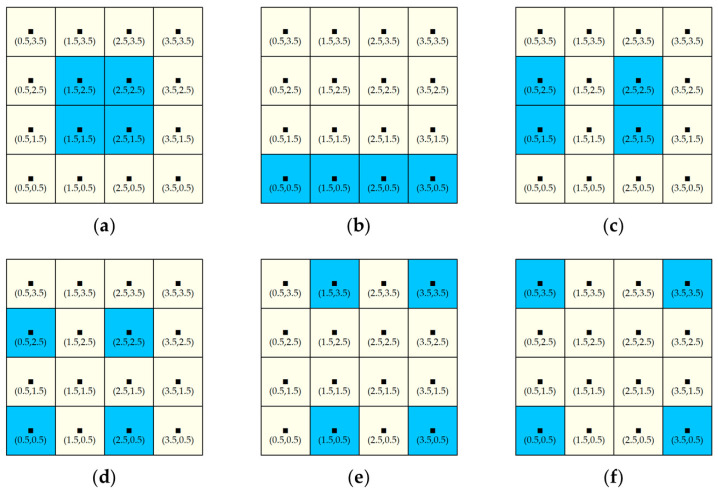
Increasing SEDUS with increasing dispersion of built-up land in constant area configurations (Pattern 1). Note: Each subplot contains 16 grid subzones or cells, where a blue grid cell (1 m × 1 m) indicates that the built-up land area in the cell is equal to the total area of the cell; each yellow cell indicates the land without buildings in it. The black spot in each grid cell indicates the geographic center of each subzone; the values inside parentheses are the relative coordinate values of its geographic center.

**Figure 7:**
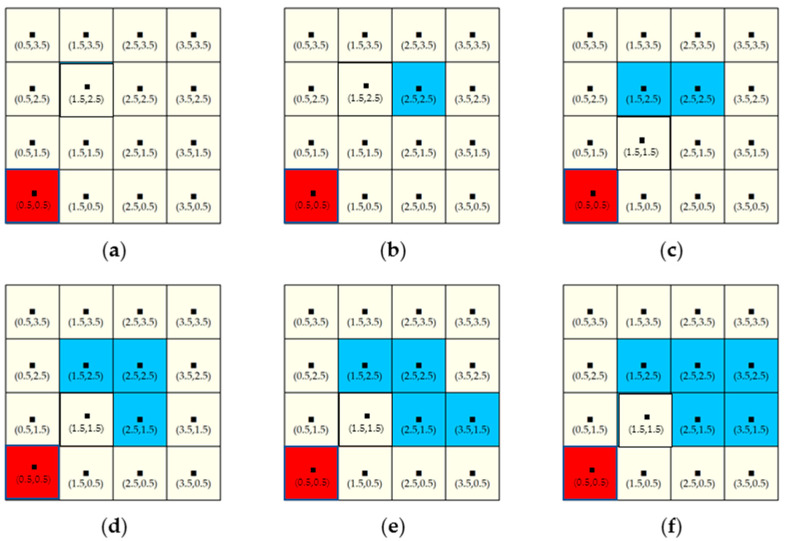
Increasing SEDUS in more balanced built-up land area configurations (Pattern 2). Note: The red grid cell can be viewed as old urban area, the blue one can be regarded as the patch of newly-grown built-up land, and (**a**–**f**) can be understood as the process of urban sprawl over time; other legends or labels are the same as in Figure 6.

For providing a clear visual effect, instead of choosing the real data of urban sprawl, we selected a grid map containing six study subplots as a study case to verify the robustness of the new index. Meanwhile, to demonstrate the effect that SEDUS changes with the two dimensions mentioned above, we presented two contrastive patterns, where Pattern 1 denotes the increasing SEDUS with increasing dispersion of built-up land in constant area configurations (Figure 6); Pattern 2 shows an increasing SEDUS in more balanced built-up land area configurations (Figure 7).

## 3. Results 

**Figure 8:**
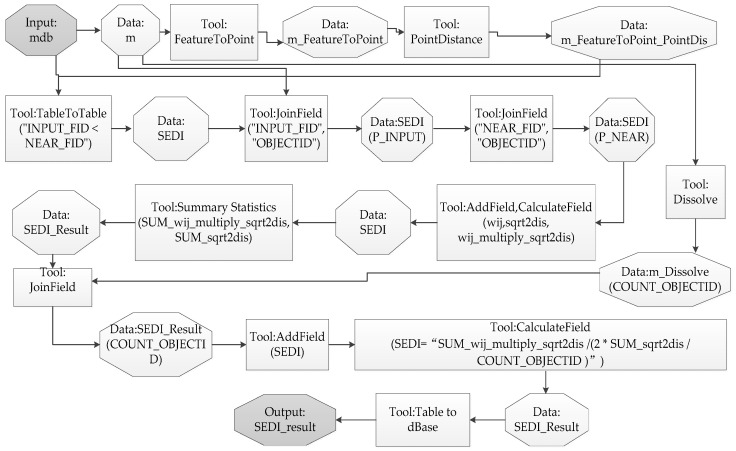
The plot of the data processing flow. Note: “P_INPUT” (or “P_NEAR”) corresponds to *p* or p* in Equations (2) and (3).

The ArcGIS 10.1 software from ESRI (Environmental Systems Research Institute, Inc. Redlands, California, USA) was employed to perform the data processing, whose flow is shown in Figure 8. In order to improve the computational efficiency and accuracy, we made a Python program (see Supplementary Materials) to perform the data processing. The resulting values of SEDI applied to Pattern 1 (Figure 6) were: (**a**) (0.1484), (**b**) (0.1759), (**c**) (0.1820), (**d**) (0.2099), (**e**) (0.2348), and (**f**) (0.2571), respectively; while the values in Pattern 2 (Figure 7) were: (**a**) (0.0000), (**b**) (0.0783), (**c**) (0.1296), (**d**) (0.1830), (**e**) (0.2481), and (**f**) (0.3076), respectively. The results matched well the tendency of urban sprawl in Pattern 1 and 2. Pattern 1 can correspond to many practical applications, for example, controlling urban sprawl requires optimizing the structure and layout of land use in the process of drawing up or amending an overall plan of land use, especially in the case of the total amount of built-up land area being constant. That is, based on resulting values of SEDI applied to different area configurations of the land use layout, a suitable plan for urban development can be chosen. Pattern 2 reproduces the phenomenon that a new town (i.e., satellite town) is emerging far away from the old central city. The new index SEDI can confirm SEDUS and provide accurate changing information of SEDUS for urban planning decision-making. Based on the information, targeted strategies of controlling urban sprawl can be proposed.

## 4. Discussion

### 4.1. Comparsion with Commonly Used Indices of SEDUS

We selected the patch shape index (PSI) from the landscape metrics [55], the Geary coefficient (GI) [20], the relative Shannon entropy index (RS) [29], and the leapfrog index (LPI) [31] to perform their capability to compare with the new index, because they are the most widely used in measuring SEDUS [6,20,29,31,55,56]. Their mathematical expressions and meanings are listed in Table 1. Applying these indices to Figure 6 and Figure 7, we recorded the resulting values in Table 2 and Table 3, respectively. As in Table 2 and Table 3, the values of PSI and GI show fluctuation trends, which do not match SEDUS in Figure 6 and Figure 7, so, we can conclude that the two indices are not suitable to characterize SEDUS. At the same time, we can see that RS and LPI can confirm the gradual increasing SEDUS in Figure 7, while the two indices cannot identify the trends in Figure 6. Since Pattern 2 (Figure 7) represents a commonplace urban sprawl pattern, which misleads us with the illusion that both indices, especially RS, can confirm SEDUS of any urban sprawl patterns. As in Table 2 and Table 3, the new index, SEDI, could confirm the two patterns, so, the new index has an apparent advantage in measuring SEDUS. 

### 4.2. The Extreme Value and Certainty of SEDI

Dietzel et al. empirically tested a theory of spatiotemporal urban growth dynamics, which suggests that the patterns of SEDUS can be characterized into two types: diffusion and compaction, respectively [57]. This theory also suggests that the processes is continuously observable until the geographical area becomes completely urbanized. Here, we can regard diffusion pattern as the maximum of spatial expansion degree, and compaction pattern as the minimum. If the maximum and the minimum are mapped as integer 1 and integer 0, the changes in the value of SEDI can be understood as the dynamic change between the two states of diffusion and compaction. Each different value of SEDI can indicate a certain spatial expansion degree. If there is no extreme value, there is no standard to quantitatively describe spatial expansion degree, so the extreme value of SEDI is the qualitative prerequisite for the certainty of SEDI. 

### 4.3. The New Index Deepening the Understanding of the Concept of Second-Order Metrics

The new measure SEDI belongs to the second-order metrics of point pattern analysis. The second-order metrics, such as F(d) function, G(d) function, and Ripley’s K function [51], characterize the general relationship of homogeneous point-pairs (homogeneous point-pairs can be understood as each point in a point-pair with a same size). The new index can examine the relationship of heterogeneous point-pairs, which greatly deepens the understanding of the concept of second-order metrics. In other words, conventional point-pairs can be taken as a special case of point-pair entropy in which each point has an equal area weight.

### 4.4. Effect of Partitioning Method on SEDI

**Figure 9:**
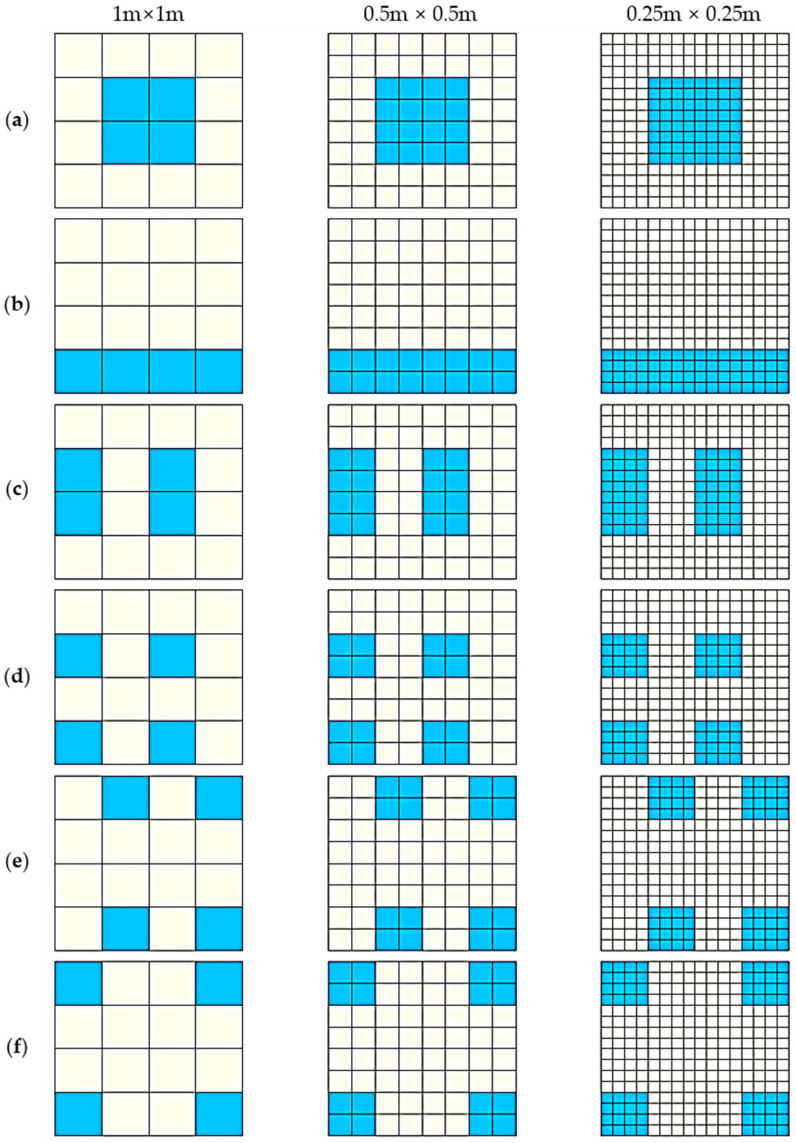
SEDUS in different scenarios at different scales. Note: From the vertical configurations in the figure, the SEDUS from (**a**) to (**f**) is gradually deepening; the horizontal subplots indicate the same configurations at three scales. The legends or labels in the figure are same as in Figure 6.

Like most spatial metrics, the new index is also influenced by the scaling problem. Here, we demonstrate the scale effect by comparing the values of SEDI in different scenarios at three scales (Figure 9). 

From Table 4, we can see that, given a fixed extent, as for the same scenario and different scales, the smaller the scale is, the greater the value of SEDI. However, as can be seen from Table 4, although the values of SEDI varied with different scales, the relative differences are small.

### 4.5. Relativity of SEDI in Measuring Urban Sprawl

As the literature states, if only considering urban sprawl as a typical geographical phenomenon, measuring urban sprawl requires two dimensions: the increasing amount of built-up land area and spatial expansion degree [7,21]. Meanwhile, from the study described above, measuring the spatial expansion degree also requires two dimensions the distances between point-pairs and area configurations, respectively. The new index can confirm well the spatial expansion degree, but it is only one side in measuring urban sprawl. The reason is that the index of SEDI based on “*p*” is a relative numerical indicator and “increasing amount of built-up land area” is an absolute one. The same thing applies to the index of RS. 

### 4.6. Future Work

In the next step, the new index can be used individually or in combination with other metrics to analyze a region in different periods, or different regions in the same period. From a broader perspective, the new index cannot just limit to urban sprawl research, but can be extended to other spatial differentiation research, such as arable land distribution and the spread of infectious diseases. 

## 5. Conclusions

Urban sprawl can result in excessive resource consumption, environmental pollution, and ecosystem destruction, which has received much attention and has become an important research topic. Accurately confirming the spatial expansion degree of urban sprawl (SEDUS) will not only provide monitoring information of land use/cover change for government officials and planners to propose targeted strategies of controlling urban sprawl, but also make contributions to the protection of the environment and ecosystems, thus, it has important theoretical and practical significance. Until now, about 50 metrics used to measure SEDUS have been proposed, however, there is no reliable metric among them. In this paper, and through previous work, we proposed developing a new index named the spatial expansion degree index (SEDI), to overcome the difficulty. 

Based on the literature, this paper articulated that, regardless of driving forces of urban sprawl, SEDUS changes with two dimensions: the distances between linked subzones and built-up land area configurations, respectively, and confirmed that SEDUS is a function of the two dimensions. We utilized Pythagoras’ theorem to measure the magnitude of the different distances, employed binary entropy function to measure the other’s dimension, and used the product of the two dimensions to characterize the comprehensive effect of SEDUS. Meanwhile, the study adopted the second-order metrics of point pattern analysis as spatial analysis method, followed the research approach from a single point-pair to multiple point-pairs and, finally, the new spatial expansion degree index (SEDI) was used to accurately confirm that SEDUS was successfully built up.

This study shows that the new index is capable of identifying SEDUS in constant or changing built-up land area configurations. Meanwhile, we also found that the patch shape index (PSI) and the Geary coefficient (GI) are not suitable to characterize SEDUS, and the relative Shannon entropy index (RS) and leapfrog index (LPI) could identify the SEDUS in the changing area configurations, but they could not identify SEDUS in the constant area configurations. Therefore, the new index has an obvious advantage in measuring SEDUS.

The new index is a new addition to existing second-order metrics for point pattern analysis, and it has three excellent characteristics: extreme value, certainty, and point-pair heterogeneity. The extreme value is the key basis of quantitative SEDUS. The values of the index range from the minimum 0 to the maximum 1, and the greater the value is, the greater the SEDUS. Thus, if the extreme values are mapped as integer 1 and integer 0, the changes in the value of SEDI can be understood as the dynamic change between the two extreme states, that is, SEDUS has quantitative uncertainty. The point-pair heterogeneity of the new index, which is mainly reflected in binary entropy function, greatly deepening the understanding of the concept of the point-pair from homogeneity to heterogeneity. 

In the next step, the new index can be applied to practical applications, such as measuring SEDUS or amending an overall plan of land use. From a broader perspective, the new index can be applied to other different spatial differentiation studies, such as arable land distribution and the spread of infectious diseases. Due to the index of SEDI being a relative numerical indicator, it has relativity in measuring urban sprawl. Meanwhile, this study shows that the new index is still influenced by partition schemes. However, given the same partition scheme in the research process, the new index is considered as a robust tool in measuring SEDUS for government officials and relevant theoretical research workers. 

## Figures and Tables

**Table 1:** The mathematical expressions and meanings of the commonly used indices of SEDUS.

Index	Mathematical Expression	Explanation
**PSI**	$PSI=P/4\sqrt{A}$	*P* denotes the perimeter of the polygon measured, and *A* is its area. The greater the value, the greater the SEDUS [26].
**GI**	$GI=(N-1)\frac{\sum_{1}^{N}\sum_{1}^{N}W_{ij}{\left(X_{i}-X_{j}\right)}^{2}}{2(\sum_{1}^{N}\sum_{1}^{N}W_{ij})\sum_{1}^{N}{\left(X_{i}-X\right)}^{2}}$	*i, j* refer to different subzones, respectively; $X_{i},X_{j}$ refer to the area proportions of built-up lands of subzones *i, j* over total area, respectively; *X* is the mean value of all subzones; $W_{ij}$ is the reciprocal of the distance between the centroids of subzones *i, j.* The lower the value of GI is, the higher the SEDUS [16].
**RS**	$RS=-\sum_{1}^{n}p_{i}\log_{2}(p_{i})/\log_{2}n$	$p_{i}$ is the proportion of different land use in grid cells, and *n* is the total number of subzones. Values of the index range from 0 to 1. The greater the value, the greater the SEDUS [3].
**LPI**	$LPI=A_{out}/A_{u}$	$A_{out}$ denotes “leapfrog area”, $A_{u}$ is the total area of built-up land. The higher the value, the greater the SEDUS [20].

**Table 2:** Capability comparison of the new index and other commonly used indices in constant built-up land area configurations.

Indices	The Resulting Values of the Different Scenarios in Figure 6					
	Subplot (a)	Subplot (b)	Subplot (c)	Subplot (d)	Subplot (e)	Subplot (f)
**PSI**	1.0000	1.2500	1.5000	2.0000	2.0000	2.0000
**GI**	1.0770	0.8203	1.0697	1.0493	0.9768	0.9108
**RS**	0.5000	0.5000	0.5000	0.5000	0.5000	0.5000
**LPI**	1.0000	1.0000	1.0000	1.0000	1.0000	1.0000
**SEDI**	0.1484	0.1759	0.1820	0.2099	0.2348	0.2571

**Table 3:** Capability comparison of the new index and other commonly used indices in changing built-up land area configurations.

Indices	The Resulting Values of the Different Scenarios in Figure 7					
	Subplot (a)	Subplot (b)	Subplot (c)	Subplot (d)	Subplot (e)	Subplot (f)
**PSI**	1.0000	1.4142	1.4434	1.5000	1.5652	1.4289
**GI**	0.8356	1.0364	1.0669	1.0768	1.0501	1.0036
**RS**	0.0000	0.2500	0.3962	0.5000	0.5805	0.6462
**LPI**	0.0000	0.5000	0.6667	0.7500	0.8000	0.8333
**SEDI**	0.0000	0.0783	0.1296	0.1830	0.2481	0.3076

**Table 4:** The values of SEDI in different scenarios at different scales.

Scale	Subplot (a)	Subplot (b)	Subplot (c)	Subplot (d)	Subplot (e)	Subplot (f)
**1 m × 1 m**	0.1484	0.1759	0.1820	0.2099	0.2348	0.2571
**0. 5 m × 0.5 m**	0.1714	0.1976	0.2032	0.2296	0.2536	0.2750
**0.25 m × 0.25 m**	0.1757	0.2016	0.2070	0.2332	0.2570	0.2783

## Supplementary Materials

The following are available online at http://www.mdpi.com/1099-4300/20/8/559/s1.
