# Supplementary material for: A Novel Index Based on Binary Entropy to Confirm the Spatial Expansion Degree of Urban Sprawl
Source: Entropy (Basel). 2018 Jul 27;20(8):559. doi: 10.3390/e20080559 (PMC7513086; doi:10.3390/e20080559)
Supplement: Supplementary file 1 [file entropy-20-00559-s001.zip › data and code/Python Program/Instruction Manual of the tool.docx]

**1. Objective**

Calculate the values of SEDI

**2. Data preparations**

**1.1** Build *mdb*, and name the layer *m* (*unique name*) as follows:


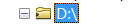


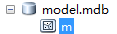


**1.2** The layer *m* must include Field *P*, and ∑*P* =1, *e.g.,* Figure 6 of the paper:


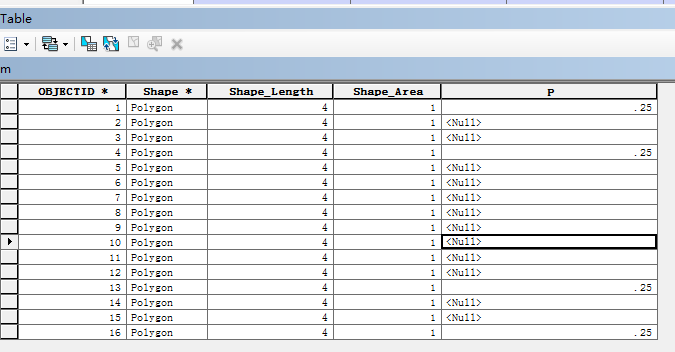


**1.3** Build a new folder to record the resulting values of the index：


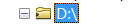


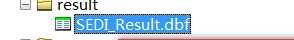


**3.Running codes**

Please open Python codes，modify the input and output parameters:


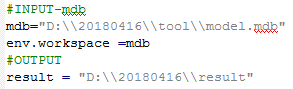


Using Python IDLE to open the code，press " F5"; wait for a while, then the resulting values of the index can be produced：


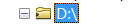


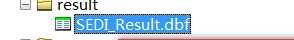


Notes: during the operation of the program, the ARCGIS Software should be shut down. Before applying the program to another layer, all files under the "*result*" layer must be removed; the name of the new layer should be replaced with ''*m*''.

*Good luck!*
